# Supplementary material for: Discrepancies between pre-specified and reported primary outcomes: A cross-sectional analysis of randomized controlled trials in gastroenterology and hepatology journals
Source: PLoS One. 2024 Nov 22;19(11):e0305027. doi: 10.1371/journal.pone.0305027 (PMC11584078; doi:10.1371/journal.pone.0305027)
Supplement: S1 File — (DOCX) [file pone.0305027.s001.docx]

**S1 File. Search Strategy**

|  | **Search strategy** |
| --- | --- |
| **#1** | "Random Allocation"[MeSH Terms] OR "Controlled Clinical Trials as Topic"[MeSH Terms] OR "Controlled Clinical Trial"[Publication Type] OR "Randomized Controlled Trials as Topic"[MeSH Terms] OR "Randomized Controlled Trial"[Publication Type] OR "Clinical Trials as Topic"[MeSH Terms] OR ("Random Allocation"[Title/Abstract] OR "Controlled Clinical Trial"[Title/Abstract] OR "Randomized Controlled Trial"[Title/Abstract] OR "RCT"[Title/Abstract] OR "clinical trial randomized"[Title/Abstract] OR (("Clinical Trials as Topic"[MeSH Terms] OR ("Clinical"[All Fields] AND "trials"[All Fields] AND "topic"[All Fields]) OR "Clinical Trials as Topic"[All Fields] OR "Trial"[All Fields] OR "trial s"[All Fields] OR "trialed"[All Fields] OR "trialing"[All Fields] OR "trials"[All Fields]) AND "randomized clinical"[Title/Abstract]) OR ("Controlled"[All Fields] AND "clinical trial randomized"[Title/Abstract]) OR "allocation random"[Title/Abstract] OR "random*"[Title/Abstract] OR "control*"[Title/Abstract] OR "clinical trial"[Title/Abstract] OR "Trial"[Title/Abstract] OR "controlled trial"[Title/Abstract]) |
| **#2** | "Journal of Hepatology"[Journal] OR "Gut"[Journal] OR "Gastroenterology"[Journal] OR "Hepatology International"[Journal] OR "liver Int"[Journal] OR "LIVER TRANSPL"[Journal] OR "expert review of gastroenterology hepatology"[Journal] OR "Colorectal Dis"[Journal] OR "Techniques in Coloproctology"[Journal] OR "Journal of Gastrointestinal Oncology"[Journal] OR "Journal of Pediatric Gastroenterology and Nutrition"[Journal] OR "Digestive Surgery"[Journal] |
| **#3** | ("2017/1/1"[Date - Publication]: "2021/12/31"[Date - Publication]) |
| **#4** | #1 AND #2 AND #3 |
