# Supplementary material for: Discrepancies between pre-specified and reported primary outcomes: A cross-sectional analysis of randomized controlled trials in gastroenterology and hepatology journals
Source: PLoS One. 2024 Nov 22;19(11):e0305027. doi: 10.1371/journal.pone.0305027 (PMC11584078; doi:10.1371/journal.pone.0305027)
Supplement: S3 File — (DOCX) [file pone.0305027.s003.docx]

**S3 File. Data Extraction Form**

| **Journal Information** |
| --- |
| 1. Journal impact factor |
| 2. Quartile (Quartile1, Quartile 2, Quartile 3, Quartile 4) |
| 3. Endorsement of CONSORT by journals |
| 4. Whether registration was mandatory or not |
| 5. Whether journals were a ICMJE member or endorsed by ICMJE |
| **Characteristics of Articles** |
| 1. No. of trial |
| 2. Year of publication |
| 3. Publication title |
| 4. Name of the journal |
| 5. Quartile (Quartile1, Quartile 2, Quartile 3, Quartile 4) |
| 6. Mandatory RCT registration (Yes, No) |
| 7. Country of origin of the first author |
| 8. Phase of study (I, II, III, IV, none/ not reported) |
| 9. Sample size |
| 10. Study centers (single center, multicenter, not specified) |
| 11. Source of funding (industry/ corporate, nonprofit (government / academic organizations/ hospitals/ philanthropic), multiple sources of funding, and none / not reported) |
| 12. Study outcome (efficiency/ tolerance/safety, toxicity/harm) |
| 13. Study design (superiority, equivalent, non-inferiority) |
| 14. CONSORT endorsed by authors (Yes, No) |
| **Registry Information and Discrepancies of Primary Outcomes** |
| 1. Type of trial registration (prospective, retrospective or no registration) |
| 2. Registration number |
| 3. Explicitly reported primary outcome (Yes, No) |
| 4. Primary outcome of registration |
| 5. Secondary outcome of registration |
| 6. Primary outcome of publication |
| 7. Secondary outcome of publication |
| 8. Types of discrepancies  *. No discrepancy. a. The registered primary outcome was reported as a secondary outcome in the published article.  b. The registered primary outcome was omitted in the published report.  c. A new primary outcome was introduced in the published article.  d. The published primary outcome was described as a secondary outcome in the registry.  e. The timing of the assessment of primary outcomes differed between the registered and published data. |
| 9. Type of publication bias  *. No publication bias. a. A new statistically significant primary outcome was introduced in the published articles. b. Nonsignificant primary outcome was omitted or defined as nonprimary in the published articles. c. Registered statistically significant secondary outcomes became published primary outcomes. |
