# Supplementary material for: Discrepancies between pre-specified and reported primary outcomes: A cross-sectional analysis of randomized controlled trials in gastroenterology and hepatology journals
Source: PLoS One. 2024 Nov 22;19(11):e0305027. doi: 10.1371/journal.pone.0305027 (PMC11584078; doi:10.1371/journal.pone.0305027)
Supplement: Attachment — Submitted filename: PONE-S-24-26486-R1-Response to reviewers.docx [file pone.0305027.s005.docx]

**ID:** **PONE-S-24-26486**

**Manuscript:** **Discrepancies between pre-specified and reported primary outcomes: a cross-sectional analysis of randomized controlled trials in gastroenterology and hepatology journals**

Dear Academic Editor Sascha Köpke, Dr. Martin Holst (Reviewer #1) and Reviewer #2,

We are very grateful to you for the constructive comments on our paper, which have substantially improved the overall quality of this manuscript. All the comments have been carefully reviewed and addressed, with corresponding changes denoted in the manuscript. A clean, unannotated copy of the revised paper is also provided for your review. Additionally, we have provided further explanations where needed. We believe these revisions have strengthened the manuscript and made it more informative for our readers. Once again, thank you for your invaluable input. We look forward to your feedback on the revised manuscript.

Best Regards,

Kaiping Zhang, PhD, MPH

**Point-to-point Reply to Reviewers’ Comments**

**Reviewer #1**

Dear editor, dear authors,

Thank you for giving me the opportunity to review the paper entitled “Discrepancies between pre-specified and reported primary outcomes: a cross-sectional analysis of randomized controlled trials in gastroenterology and hepatology journals”.

The paper describes registration rates, discrepancies between pre-specified and reported primary outcomes, as well as publication and reporting bias in RCTs focusing on journals in gastroenterology and hepatology. While registration rates are found to be relatively high and stable over time, about one quarter of assessed trials contain discrepancies in their registered and reported primary outcomes, in particular differences in assessment timing. The manuscript also reports many statistical tests aimed at finding associations of these practices with trial or journal characteristics.

I would like to congratulate the authors to their well-conducted work that adds important knowledge on registration rates and outcome switching in a specific field.

**Reply:** Thank you for your positive comments and effort in reviewing our manuscript.

During review, I found a few major points that I would suggest the authors address:

**Comment 1**

First, I would like to suggest that you have a native English speaker read through the document. I am not a native speaker myself, but I got the impression that there are a few sections that could use a bit of language polishing.

**Reply:** The manuscript was proofread by three native English speakers before its submission. However, we understand that some language issues may still exist and bring potential barriers to understanding. The manuscript is now thoroughly polished by a native English speaker.

**Changes in the text:** Please see the revised manuscript.

**Comment 2**

Second, could you provide - if available - any link of reference to where your study was preregistered?

**Reply:** Thank you for your comment. We did not preregister for the study. We thought the study could be waived of mandatory registration, considering that it is a retrospective and observational study (not a clinical trial), and that the study is only based on published and open records (without retrieving any patients’ data). However, if the reviewer feels that registration is required, we would be happy to conduct a supplementary registration.

**Changes in the text:** None.

**Comment 3**

ll. 28-29:

I think the statement “However, whether similar discrepancies exist in RCTs focusing on gastrointestinal and liver diseases remains unclear” is not warranted. You yourself state that another study has already been conducted in the field. Please tone down that statement.

**Reply:** Many thanks for the suggestion. We have adjusted the statement accordingly.

**Changes in the text:** Lines 28-29 "However, studies examining whether similar discrepancies exist in RCTs focusing on gastrointestinal and liver diseases are limited ".

**Comment 4**

ll. 45.46 (and similar text passages throughout the manuscript):

I consider sentences like "There was a lower risk of primary outcome discrepancies associated with more recent publication years (OR=0.267, 46 95% CI: 0.101, 0.706, p=0.008)" in the Abstract's conclusions a bit overstretching. You report a large number of univariate analyses (I counted approximately 50). Under the circumstances, this could as well be a chance finding and should not be featured so prominently.

**Reply:** We appreciate your valuable suggestion. We apologize for any confusion. The univariate analysis included 10 variables, with the only significant difference finding related to the publication year 2021. We agree that this could be a chance finding and have revised the sentence to reflect that possibility, as well as specifying the exact year ‘2021’.

**Changes in the text:** Lines 47 "There was a lower chance of primary outcome discrepancies associated with more recent publication year (year 2021) (OR=0.267, 95% CI: 0.101, 0.706, p=0.008)."

**Comment 5**

ll. 114-118:

I would like to better understand the reasoning how the journals were chosen. Why did you choose the "top" journals in each quartile, as oppopsed to, for example, a random sample from each quartile or a random sample overall? I am sure you have very good reasons for your choice, but more detail would help readers understand this better.

**Reply:** Thank you for your insightful comment. We agree that including journals via a random sample could provide a broader perspective. However, we selected the "top" journals from each quartile to better address our initial research hypothesis and question. We initially hypothesized that RCTs published in higher-ranked journals may demonstrate better consistency in primary outcome reporting, so we stratified our selection by quartile. Additionally, we aimed to assess primary outcome consistency specifically within the “top” journals of each quartile. Thus, the "top" concept encompasses both between and within quartile considerations. Interestingly, as shown in table 5, we found no significant differences between different quartiles of journals. We acknowledge your point and have provided further details for clarity.

**Changes in the text:** Lines 343-347 "Our study diverges from prior research by focusing on top journals within different quartiles. Our initial hypothesis was that top journals in the higher-ranked quartile would exhibit fewer discrepancies in trial reporting. Contrary to expectations, we found no significant differences in the risk of outcome reporting bias based on the journal’s impact-factor quartile, suggesting that quartile may not reliably indicate reporting consistency".

**Comment 6**

l. 166:

Sentence "or if a nonsignificant primary outcome was omitted”. I am still struggling to understand how you can assess this. If the outcome was omitted, there is no way of telling whether it was significant or non-significant, right? Please explain this in a bit more detail.

**Reply:** Thank you for your insightful question. You are correct that if an outcome is omitted, we cannot directly determine whether it was significant or non-significant. Therefore, we followed the approach outlined by Chan AW et al. (JAMA 2004;291(20):2457-2465. PMID: 15161896), where an omitted primary outcome is defined as one listed in the registration but missing from the published article with no reported results. A reasonable assumption is that the omitted outcome might not have achieved statistical significance, leading to its exclusion from the published results. This assumption is based on the notion that statistically significant results are more likely to be reported and published due to the well-known publication bias. Regretfully,

no one else can directly assess its significance (except the authors themselves). We hope this clarifies our methodology.

**Changes in the text:** None.

**Comment 7**

Table 3: I don’t think this Table works as it is. Although it is very concise (which was probably your intention), I think it will be rather confusing to readers because the numbers do not add up. My suggestion is that you make three different tables, with: Table 3a - Column 1: Registered trials, Column 2: Unregistered trials, Column 3: Total of all trials. Table 3b - Column 1: Trials with prospective registration, Column 2: Trials with retrospective registration, Column 3: Total of all registered trials. Table 3c - Column 1: Trials with primary outcome agreement, Column 2: Trials with primary outcome disagreement, Column 3: Total of all eligible trials (i.e., all trials for which primary outcome discrepancies were assessed). Then you can present the P-Values at the right side (similar to Table 4), so it becomes clear which groups were compared. I know this leads to more and larger tables, but I think they will be much more understandable. Of course, feel free to choose a different presentation if you have a better idea.

**Reply:** We appreciate this informative comment and have revised Table 3 as suggested.

**Changes in the text:** Please see the revised Table 3.

Table 3. Registration, prospective registration, and primary outcome agreement across clinical trials, stratified by trial characteristics

| **Table 3a** | | | | |
| --- | --- | --- | --- | --- |
| **Feature** | **Total (n=362)** | **Registered trials (n=312)** | **Unregistered trials(n=50)** | **P-Value** |
| **Year of publication, n (%)** |  |  |  | 0.83 |
| 2017 | 73 | 63(86.3) | 10(13.7) |  |
| 2018 | 64 | 52(81.3) | 12(18.7) |  |
| 2019 | 82 | 76(92.7) | 6(7.3) |  |
| 2020 | 75 | 61(81.3) | 14(18.7) |  |
| 2021 | 68 | 60(88.2) | 8(11.8) |  |
| **Study Location, n (%)** |  |  |  |  |
| Europe | 148 | 131(88.5) | 17(11.5) | 0.009 * |
| America | 100 | 91(91.0) | 9(9.0) |  |
| Asia | 99 | 78(78.8) | 21(21.2) |  |
| Oceania | 9 | 9(100.0) | 0(0.0) |  |
| Africa | 6 | 3(50.0) | 3(50.0) |  |
| **Quartile, n (%)** |  |  |  | <0.001* |
| Q1 | 197 | 185(93.9) | 12(6.1) |  |
| Q2 | 52 | 39(75.0) | 13(25.0) |  |
| Q3 | 50 | 39(78.0) | 11(22.0) |  |
| Q4 | 63 | 49(77.8) | 14(22.2) |  |
| **Funding, n (%)** |  |  |  | 0.01* |
| Industry | 109 | 97(89.0) | 12(11.0) |  |
| Nonprofit | 156 | 139(89.1) | 17(10.9) |  |
| Mixed | 17 | 16(94.1) | 1(5.9) |  |
| None disclosed/no funding | 80 | 60(75.0) | 20(25.0) |  |
| **Trial design, n (%)** |  |  |  | 0.21 |
| Superiority | 338 | 289(85.5) | 49(14.5) |  |
| Equivalency | 4 | 4(100.0) | 0(0.0) |  |
| Noninferiority | 20 | 19(95.0) | 1(5.0) |  |

| **Table 3b** | | | | | | |
| --- | --- | --- | --- | --- | --- | --- |
| **Feature** | **Total (n=312)** | **Prospective Registration (n=249)** | | **Retrospective Registration (n=63)** | | **P-value** |
| **Year of publication, n (%)** |  |  | |  | | 0.92 |
| 2017 | 63 | 51(81.0) | | 12(19.0) | |  |
| 2018 | 52 | 42(80.8) | | 10(19.2) | |  |
| 2019 | 76 | 57(75.0) | | 19(25.0) | |  |
| 2020 | 61 | 53(86.9) | | 8(13.1) | |  |
| 2021 | 60 | 46(76.7) | | 14(23.3) | |  |
| **Study Location, n (%)** |  |  | |  | | 0.74 |
| Europe | 131 | 105(80.2) | | 26(19.8) | |  |
| America | 91 | 76(83.5) | | 15(16.5) | |  |
| Asia | 78 | 59(75.6) | | 19(24.4) | |  |
| Oceania | 9 | 7(77.8) | | 2(22.2) | |  |
| Africa | 3 | 2(66.7) | | 1(33.3) | |  |
| **Quartile, n (%)** |  |  | |  | | <0.001* |
| Q1 | 185 | 163(88.1) | | 22(11.9) | |  |
| Q2 | 39 | 25(64.1) | | 14(35.9) | |  |
| Q3 | 39 | 31(79.5) | | 8(20.5) | |  |
| Q4 | 49 | 30(61.2) | | 19(38.8) | |  |
| **Funding, n (%)** |  |  | |  | | 0.001* |
| Industry | 97 | 90(92.8) | | 7(7.2) | |  |
| Nonprofit | 139 | 102(73.4) | | 37(26.6) | |  |
| Mixed | 16 | 14(87.5) | | 2(12.5) | |  |
| None disclosed/no funding | 60 | 43(71.7) | | 17(28.3) | |  |
| **Trial design, n (%)** |  |  | |  | | 0.006* |
| Superiority | 289 | 230(79.6) | | 59(20.4) | |  |
| Equivalency | 4 | 1(25.0) | | 3(75.0) | |  |
| Noninferiority | 19 | 18(94.7) | | 1(5.3) | |  |
| **Table 3c** | | | | | | |
| **Feature** | **Total (n=285)** | | **Primary-Outcome Agreement Between Registry and Publication (n=209)** | **Primary-Outcome Disagreement Between Registry and Publication (n=76)** | **P-value** | |
| **Year of publication, n (%)** |  | |  |  | 0.53 | |
| 2017 | 56 | | 37(66.1) | 19(33.9) |  | |
| 2018 | 50 | | 39(78.0) | 11(22.0) |  | |
| 2019 | 68 | | 47(69.1) | 21(30.9) |  | |
| 2020 | 58 | | 51(87.9) | 7(12.1) |  | |
| 2021 | 53 | | 35(66.0) | 18(34.0) |  | |
| **Study Location, n (%)** |  | |  |  | 0.51 | |
| Europe | 120 | | 85(70.8) | 35(29.2) |  | |
| America | 82 | | 65(79.3) | 17(20.7) |  | |
| Asia | 72 | | 50(69.4) | 22(30.6) |  | |
| Oceania | 8 | | 7(87.5) | 1(12.5) |  | |
| Africa | 3 | | 2(66.7) | 1(33.3) |  | |
| **Quartile, n (%)** |  | |  |  | 0.17 | |
| Q1 | 174 | | 134(77.0) | 40(23.0) |  | |
| Q2 | 34 | | 21(61.8) | 13(38.2) |  | |
| Q3 | 36 | | 27(75.0) | 9(25.0) |  | |
| Q4 | 41 | | 27(65.9) | 14(34.1) |  | |
| **Funding, n (%)** |  | |  |  | 0.18 | |
| Industry | 94 | | 76(80.9) | 18(19.1) |  | |
| Nonprofit | 128 | | 88(68.8) | 40(31.2) |  | |
| Mixed | 11 | | 9(81.8) | 2(18.2) |  | |
| None disclosed/no funding | 52 | | 36(69.2) | 16(30.8) |  | |
| **Trial design, n (%)** |  | |  |  | 0.29 | |
| Superiority | 264 | | 191(72.3) | 73(27.7) |  | |
| Equivalency | 3 | | 3(100.0) | 0(0.0) |  | |
| Noninferiority | 18 | | 15(83.3) | 3(16.7) |  | |

Q: Quartile.

*: p< 0.05.

**Comment 8**

Figure 1: The replacement of journals is not included in the flowchart. If one took it literally, one would expect that only trials from 10 journals were included.

**Reply:** Thank you for this important comment. We have now included the process of replacing the two journals.

**Changes in the text:** Please see revised Figure 1.

**Comment 9**

Figure 2A:

For the Table on the right, the year should rather be the top row, not the bottom row.

**Reply:** Many thanks for the suggestion. Considering that the revised Table 3 contains repeated information in the previous Figure 2, we now have deleted Figure 2.

**Changes in the text:** Figure 2 is deleted. Please see the revised Table 3.

**Comment 10**

Short title: The short title reads “Discrepancies between registered and reported primary outcome” - it probably should be „primary outcomes ?

**Reply:** We apologize for this mistake and have changed it.

**Changes in the text:** Short title: "Discrepancies Between Registered and Reported Primary Outcomes"

**Comment 11**

l. 38: I suggest you do not list all the statistical tests and models used, but instead, only the tests that are reported in the Abstract’s results section (which I understand is only the logistic regression model).

**Reply:** Thank you for your valuable feedback. We understand your suggestion to list only the statistical tests and models reported in the Abstract's results section. However, we believe it is important to mention all statistical analysis methods in the Abstract's methods section to provide a complete overview of our methodology used. Therefore, we have decided to retain this information to ensures clarity and comprehensiveness for readers.

**Changes in the text**: None.

**Comment 12**

l. 45: (and other parts in the text): Please rather write "lower chance “instead of "lower risk”. I think "risk" has too much of an accusing tone in this context.

**Reply:** Thank you for your advice. We have reviewed the full text and made revisions accordingly.

**Changes in the text:** Line 47 "There was a lower chance of primary outcome discrepancies associated with more recent publication years (Year 2021) (OR=0.267, 95% CI: 0.101, 0.706, p=0.008)". And Lines 288-289 "A more recent publication year was associated with a lower chance of primary outcome discrepancy".

**Comment 13**

ll. 47-48: Regarding the non-significant influences, please also consistently report ORs and CIs.

**Reply:** Many thanks for the suggestion. We conducted the chi-square test to assess the relationship between journal quartiles and both primary outcome consistency and potential reporting bias. We want to clarify that in the context of chi-square tests, it is standard practice to report p-values to indicate statistical significance. Including ORs and CIs for non-significant results may not provide additional meaningful information. We hope this clarifies our reporting approach.

**Changes in the text:** None.

**Comment 14**

ll. 77-79A: Please provide the original citation for this finding, not another paper that cited the original source. If possible, I’d also suggest you report a median instead of a mean, with no decimal places.

**Reply:** Thank you for your insightful feedback. We acknowledge the importance of citing the original source and revised our citation accordingly. Regarding the reporting of median values, we reviewed the original source and found that only mean value were reported, specifically "On average, each trial reported just 58.2% of its specified outcomes. And on average, each trial silently added 5.3 new outcomes." Therefore, we have retained the mean values in our article.

**Changes in the text:** Reference 6: Goldacre B, Drysdale H, Smith AP, Dale A, Milosevic A, Slade E, et al. The COMPare Trials project. Available from: https://www.compare-trials.org/

**Comment 15**

ll.129-130: I don’t understand why you did not include crossover trials? Are they not a type of randomized-controlled trial? (This could be a misunderstanding from my side, of course.)

**Reply:** Thank you for your thoughtful feedback. We acknowledge that crossover trials are indeed a type of RCT. However, the primary objective of our study was to evaluate the consistency between registered and published outcomes in parallel-group RCTs comparing two different treatment modalities. While valuable, crossover trials involve a different design where the same participants receive treatments sequentially, which introduces unique complexities and potential carryover effects. These factors make crossover trials less comparable to parallel-group RCTs for our specific analysis. Therefore, for methodological consistency and clarity, we chose to focus exclusively on parallel-group RCTs. This approach aligns with other studies that have a similar research focus. For instance,

(1) Won J, Kim S, Bae I, Lee H. Trial registration as a safeguard against outcome reporting bias and spin? A case study of randomized controlled trials of acupuncture. PLoS One. 2019 Oct 3;14(10):e0223305. doi: 10.1371/journal.pone.0223305.

(2) Smith SM, Wang AT, Pereira A, et al. Discrepancies between registered and published primary outcome specifications in analgesic trials: ACTTION systematic review and recommendations. Pain. 2013 Dec;154(12):2769-2774.

**Changes in the text:** Lines 126-128 "We excluded reviews, observational studies, systematic reviews or meta-analyses, cost-effectiveness analyses, animal or in vitro studies, case reports, cross-over studies, as well as ancillary studies (e.g., protocol studies, secondary analyses, follow-up studies, subgroup analyses, and post hoc analyses)".

**Comment 16**

ll. 130-131: Can you provide more detail on how exactly you searched for the full text?

**Reply:** Thank you for your comments. We employed a comprehensive approach to download the full texts of eligible articles. Initially, we accessed free articles directly through open-access journals. For articles that were not freely available, we conducted further searches in various databases to which we have subscriptions, such as Web of Science, Embase, and Scopus. These databases provide extensive coverage and access to a wide range of journal articles. There was only one paper for which we could not obtain the full text.

**Changes in the text:** Lines 128-130 “We accessed free articles directly through open-access journals. For articles that were not freely available, we thoroughly searched the full text of each article using multiple sources, including Web of Science, Embase, and Scopus, among others.

**Comment 17**

l. 137: Rather “resolved through discussion “instead of “resolved through consensus”?

**Reply:** We apologize for not explaining it clearly. Disagreements were resolved through discussion until a consensus was reached.

**Changes in the text:** Lines 136-137 "Any disagreements were resolved through discussion until a consensus was reached". And Line 143 "Any conflicts were resolved through discussion with a third reviewer (FHY) until a consensus was reached ".

**Comment 18**

l. 147-148: “If the registry number was uncovered in this way, the trial was deemed unregistered”. Can you provide a reasoning why?

**Reply:** Thank you for your insightful question. We adopted a thorough approach inspired by Killeen et al, (1) and Rankin et al, (2) to ensure that we did not miss any registration numbers. Specifically, we undertook the following steps to identify trial registry numbers: 1. Verification through the WHO International Clinical Trials Registry Platform (ICTRP): This platform consolidates trial registrations from various registries worldwide, providing a comprehensive resource for verifying the validity of registry numbers. 2. Supplementary Searches: In cases where the registry number was not immediately apparent, we conducted additional searches based on the trial's publication title, authors' names, trial participants, and primary sponsors. This supplementary search aimed to cross-reference and uncover any potential registrations that might have been missed initially. If a trial's registry number could not be identified through these extensive efforts, we deemed the trial unregistered. This approach ensures that we are as exhaustive and accurate as possible in our identification process, thereby maintaining the reliability of our methodology.

References:

(1) Killeen S, Sourallous P, Hunter IA, Hartley JE, Grady HL. Registration rates, adequacy of registration, and a comparison of registered and published primary outcomes in randomized controlled trials published in surgery journals. Ann Surg. 2014 Jan;259(1):193-6.

(2) Rankin J, Ross A, Baker J, O'Brien M, Scheckel C, Vassar M. Selective outcome reporting in obesity clinical trials: a cross-sectional review. Clin Obes. 2017 Aug;7(4):245-254.

**Changes in the text:** None.

**Comment 19**

ll. 169-170: How are “initially registered” primary outcomes defined? As the first registry entry at all? Or the first registry entry after study start? Did all of the included registries actually allow for an assessment of historical versions? Please provide more detail on these questions.

**Reply:** We apologize for the confusion and have made additions to address the comment. The "initially registered" primary outcomes were defined as those listed in the first registry entry available on the WHO International Clinical Trials Registry Platform. These outcomes are the ones specified at the time of the initial registration, and do not include any outcomes added or modified in subsequent updates. While the ICTRP provides a comprehensive registry, it does not always allow for a clear assessment of historical versions or the exact trial stage at which changes were made. For instance, prospective registrations may have retrospective changes where authors modify primary outcomes after concluding the trial. Despite these challenges, we did extract information on changes to primary outcomes when such data were accessible. We found that the primary outcomes were modified in 3 of the included trials. However, given the above concern, we ultimately chose to compare the initially registered primary outcomes. We hope this detailed explanation clarifies our methodology and the considerations we took into account.

**Changes in the text:** Lines 154-159 "Two investigators (BHS, FHY) compared the primary outcomes reported in the articles with those initially registered to determine consistency. These outcomes reflect those specified at the time of the initial registration and do not include any outcomes added or modified in subsequent updates. We chose these primary outcomes because, while the ICTRP provides a comprehensive registry, it does not always allow for a clear assessment of historical versions or the exact trial stage at which changes were made. For instance, prospective registrations may have retrospective changes where authors modify primary outcomes after concluding the trial."

**Comment 20**

ll. 193-196: Minor suggestion: Since the journal names are already in the table, I’d leave them out of the text.

**Reply:** Thank you for your suggestion. However, we believe that mentioning the journal names in the text provides additional context and clarity for readers, allowing them to immediately recognize the sources without referring back to the table. We hope this rationale is acceptable and appreciate your understanding.

**Changes in the text:** None.

**Comment 21**

Table 5: Please write “univariate analyses” (plural, not singular).

**Reply:** We sincerely appreciate your detailed examination and we have revised it.

**Changes in the text:** Please see Table 5, line 40, line 187, and line 260- change "Univariate analysis" to "Univariate analyses".

**Comment 22**

l. 292 (“28% to 88.4%”): Please be consistent in how many digits after the comma you report.

**Reply:** Many thanks for the correction. We have refined it.

**Changes in the text:** Line 298, change "28% to 88.4%" to "28.0% to 88.4%".

**Comment 23**

ll. 388-389: “Notably, factors such as high journal quartile, developed regions, industry funding, multiple centers, prospectively registered trials, adherence to CONSORT guidelines, and mandatory registration did not show a significant association with discrepancies in primary outcome reporting. This suggests that discrepancies in primary outcome reporting may be a prevalent issue within the gastroenterology and hepatology research community”. I don’t understand how the second sentence follows the first logically.

**Reply:** We apologize for the confusion. We understand the need for greater clarity in our Conclusions and have revised it accordingly.

**Changes in the text:** Lines 410-414 "Notably, factors such as high journal quartile, developed regions, industry funding, multiple centers, prospectively registered trials, adherence to CONSORT guidelines, and mandatory registration did not show a significant association with discrepancies in primary outcome reporting. This lack of association across various trial characteristics suggests that discrepancies in primary outcome reporting are not confined to specific types of trials or contexts, but rather, are a prevalent issue within the gastroenterology and hepatology research community".

**Reviewer #2**

First of all, the authors have to be congratulated for this interesting and extensive study. However, there are some points that need to revised, especially regarding the presentation of methods and of the results in the tables.

**Reply:** Thank you for your constructive suggestions and efforts put in reviewing our manuscript.

**Comment 1**

Abstract: Please include in the methods from where the information on prespecified primary endpoints was taken.

**Reply:** Thank you for your valuable comment. We have added the information source both in Abstract-methods and Methods.

**Changes in the text:** Lines 36-37 “Pre-specified primary outcomes were retrieved from the World Health Organization’s International Clinical Trials Registry Platform” and lines 144-146 “We utilized the World Health Organization’s International Clinical Trials Registry Platform (ICTRP), comprising 20 main trial registries globally, to identify and download registration records and to retrieve pre-specified primary outcomes of published trials.”

**Comment 2**

Please specify the “more recent publication” year in the results section.

**Reply: T**hank you for your insightful comment. We have specified "Year 2021" instead of using “more recent publication” year.

**Changes in the text:** Line 47 "There was a lower chance of primary outcome discrepancies associated with more recent publication year (year 2021) (OR=0.267, 95% CI: 0.101, 0.706, p=0.008)."

**Comment 3**

Introduction: Generally, the introduction is well written, but on line 97 there seem to be break when introducing the field of gastroenterology. Why should there be a study in this field, when we already have data across other fields? Why should findings differ in gastroenterology?

**Reply:** Thank you for your thorough review and valuable feedback. The only relevant study we identified in this field was conducted over a decade ago in 2013, and it did not compare the primary outcome discrepancies based on journal quartiles (we mentioned this point in the Discussion section). And it is essential to determine whether the situation has improved since then. Given the evolving nature of clinical research, an updated and more rigorous methodological design evaluation is necessary to understand the current state of primary outcome reporting in gastroenterology and hepatology journals. We have refined the Introduction for better clarity.

**Changes in the text:** Lines 100-104 "However, the study did not compare discrepancies based on journal quartiles. It is also crucial to assess whether the situation has improved over the past decade. Additionally, no recent study has examined the prevalence and nature of major outcome discrepancies in high-impact and lower-impact journals within this domain. Therefore, a comprehensive and updated evaluation of the consistency between registered and published primary outcomes is warranted within the context of gastrointestinal and hepatic journals."

**Comment 4**

Materials & methods: Please delete the statement regarding PRISMA (line 118) as PRISMA is, firstly, a reporting guideline and, secondly, only suitable for systematic reviews.

**Reply:** Thank you for your valuable feedback. We have deleted the statement regarding PRISMA checklist.

**Changes in the text:** Delete the sentences “We adhered to the Preferred Reporting Items for Systematic Reviews and Meta-Analyses guidelines throughout the process of identifying, screening, and including search results, to ensure that the search strategy was comprehensive, and the eligibility was plausible”.

**Comment 5**

The screening process seems a bit unclear. Did 3 investigators independently screen each title/ abstract as well as full-text? Why did you choose 3 instead of 2?

**Reply:** Thank you for bringing this to our attention. To clarify, each article was screened independently by two investigators at each stage. A total of three investigators were involved in these processes.

**Changes in the text:** Lines 134-136 "A total of three investigators (BHS, FHY, and YL) formed cross-groups in pairs, i.e., three groups. Two investigators from each group independently screened the articles for eligibility, initially by title and abstract, followed by a full-text assessment."

**Comment 6**

I was wondering whether discrepancies regarding endpoint assessment were not an own category, for instance, when instead of 2 independent investigators to assess an endpoint data from patients’ records were drawn or quality of life was measured using another instrument than prespecified in the protocol.

**Reply:** Thank you for your thoughtful comment. We appreciate your insights and have considered your suggestion carefully. However, after reviewing your comment, we still find it challenging to fully understand the proposed categorization of discrepancies. Our primary focus was to evaluate the discrepancies between registered and published primary outcomes in randomized controlled trials published in gastroenterology and hepatology journals. To achieve this, two researchers (BHS and FHY) compared the reported primary outcomes in the articles with those initially registered to determine overall consistency. We chose to focus on this comparison to assess the overall discrepancies rather than categorizing them into separate types based on the methods of endpoint assessment. Thank you once again for your valuable feedback.

**Changes in the text:** None.

**Comment 7**

Results: You write that figure 2 shows the trends (line 228), but there are no trends over the years. The figure just shows the distribution over years.

**Reply:** Thank you for your insightful feedback. We agree with you that Figure 2 shows the distribution instead of the trends. In this case, we have deleted Figure 2 as the revised Table 3 (see below, in comment 8) already contains such information.

**Changes in the text:** Figure 2 is now deleted.

**Comment 8**

I have some difficulties in understanding table 3. For instance, you write that there are differences in study location between registered and unregistered trials. However, study locations of unregistered trials are not shown in table 3. This is not only confusing for the reader (and unclear to which comparisons these p-values belong to) but also omits important results. The same is true for all other columns, as one wants to compare studies with vs. those without prospective registration, but data are not shown.

**Reply:** We appreciate this informative comment and have revised Table 3.

**Changes in the text:** Please see revised Table 3.

Table 3. Registration, prospective registration, and primary outcome agreement across clinical trials, stratified by trial characteristics

| **Table 3a** | | | | |
| --- | --- | --- | --- | --- |
| **Feature** | **Total (n=362)** | **Registered trials (n=312)** | **Unregistered trials(n=50)** | **P-Value** |
| **Year of publication, n (%)** |  |  |  | 0.83 |
| 2017 | 73 | 63(86.3) | 10(13.7) |  |
| 2018 | 64 | 52(81.3) | 12(18.7) |  |
| 2019 | 82 | 76(92.7) | 6(7.3) |  |
| 2020 | 75 | 61(81.3) | 14(18.7) |  |
| 2021 | 68 | 60(88.2) | 8(11.8) |  |
| **Study Location, n (%)** |  |  |  |  |
| Europe | 148 | 131(88.5) | 17(11.5) | 0.009 * |
| America | 100 | 91(91.0) | 9(9.0) |  |
| Asia | 99 | 78(78.8) | 21(21.2) |  |
| Oceania | 9 | 9(100.0) | 0(0.0) |  |
| Africa | 6 | 3(50.0) | 3(50.0) |  |
| **Quartile, n (%)** |  |  |  | <0.001* |
| Q1 | 197 | 185(93.9) | 12(6.1) |  |
| Q2 | 52 | 39(75.0) | 13(25.0) |  |
| Q3 | 50 | 39(78.0) | 11(22.0) |  |
| Q4 | 63 | 49(77.8) | 14(22.2) |  |
| **Funding, n (%)** |  |  |  | 0.01* |
| Industry | 109 | 97(89.0) | 12(11.0) |  |
| Nonprofit | 156 | 139(89.1) | 17(10.9) |  |
| Mixed | 17 | 16(94.1) | 1(5.9) |  |
| None disclosed/no funding | 80 | 60(75.0) | 20(25.0) |  |
| **Trial design, n (%)** |  |  |  | 0.21 |
| Superiority | 338 | 289(85.5) | 49(14.5) |  |
| Equivalency | 4 | 4(100.0) | 0(0.0) |  |
| Noninferiority | 20 | 19(95.0) | 1(5.0) |  |

| **Table 3b** | | | | | | |
| --- | --- | --- | --- | --- | --- | --- |
| **Feature** | **Total (n=312)** | **Prospective Registration (n=249)** | | **Retrospective Registration (n=63)** | | **P-value** |
| **Year of publication, n (%)** |  |  | |  | | 0.92 |
| 2017 | 63 | 51(81.0) | | 12(19.0) | |  |
| 2018 | 52 | 42(80.8) | | 10(19.2) | |  |
| 2019 | 76 | 57(75.0) | | 19(25.0) | |  |
| 2020 | 61 | 53(86.9) | | 8(13.1) | |  |
| 2021 | 60 | 46(76.7) | | 14(23.3) | |  |
| **Study Location, n (%)** |  |  | |  | | 0.74 |
| Europe | 131 | 105(80.2) | | 26(19.8) | |  |
| America | 91 | 76(83.5) | | 15(16.5) | |  |
| Asia | 78 | 59(75.6) | | 19(24.4) | |  |
| Oceania | 9 | 7(77.8) | | 2(22.2) | |  |
| Africa | 3 | 2(66.7) | | 1(33.3) | |  |
| **Quartile, n (%)** |  |  | |  | | <0.001* |
| Q1 | 185 | 163(88.1) | | 22(11.9) | |  |
| Q2 | 39 | 25(64.1) | | 14(35.9) | |  |
| Q3 | 39 | 31(79.5) | | 8(20.5) | |  |
| Q4 | 49 | 30(61.2) | | 19(38.8) | |  |
| **Funding, n (%)** |  |  | |  | | 0.001* |
| Industry | 97 | 90(92.8) | | 7(7.2) | |  |
| Nonprofit | 139 | 102(73.4) | | 37(26.6) | |  |
| Mixed | 16 | 14(87.5) | | 2(12.5) | |  |
| None disclosed/no funding | 60 | 43(71.7) | | 17(28.3) | |  |
| **Trial design, n (%)** |  |  | |  | | 0.006* |
| Superiority | 289 | 230(79.6) | | 59(20.4) | |  |
| Equivalency | 4 | 1(25.0) | | 3(75.0) | |  |
| Noninferiority | 19 | 18(94.7) | | 1(5.3) | |  |
| **Table 3c** | | | | | | |
| **Feature** | **Total (n=285)** | | **Primary-Outcome Agreement Between Registry and Publication (n=209)** | **Primary-Outcome Disagreement Between Registry and Publication (n=76)** | **P-value** | |
| **Year of publication, n (%)** |  | |  |  | 0.53 | |
| 2017 | 56 | | 37(66.1) | 19(33.9) |  | |
| 2018 | 50 | | 39(78.0) | 11(22.0) |  | |
| 2019 | 68 | | 47(69.1) | 21(30.9) |  | |
| 2020 | 58 | | 51(87.9) | 7(12.1) |  | |
| 2021 | 53 | | 35(66.0) | 18(34.0) |  | |
| **Study Location, n (%)** |  | |  |  | 0.51 | |
| Europe | 120 | | 85(70.8) | 35(29.2) |  | |
| America | 82 | | 65(79.3) | 17(20.7) |  | |
| Asia | 72 | | 50(69.4) | 22(30.6) |  | |
| Oceania | 8 | | 7(87.5) | 1(12.5) |  | |
| Africa | 3 | | 2(66.7) | 1(33.3) |  | |
| **Quartile, n (%)** |  | |  |  | 0.17 | |
| Q1 | 174 | | 134(77.0) | 40(23.0) |  | |
| Q2 | 34 | | 21(61.8) | 13(38.2) |  | |
| Q3 | 36 | | 27(75.0) | 9(25.0) |  | |
| Q4 | 41 | | 27(65.9) | 14(34.1) |  | |
| **Funding, n (%)** |  | |  |  | 0.18 | |
| Industry | 94 | | 76(80.9) | 18(19.1) |  | |
| Nonprofit | 128 | | 88(68.8) | 40(31.2) |  | |
| Mixed | 11 | | 9(81.8) | 2(18.2) |  | |
| None disclosed/no funding | 52 | | 36(69.2) | 16(30.8) |  | |
| **Trial design, n (%)** |  | |  |  | 0.29 | |
| Superiority | 264 | | 191(72.3) | 73(27.7) |  | |
| Equivalency | 3 | | 3(100.0) | 0(0.0) |  | |
| Noninferiority | 18 | | 15(83.3) | 3(16.7) |  | |

Q: Quartile.

*: p< 0.05.

**Comment 9**

You further have to think whether these proportions presented in table 3 are really necessary or whether the important information is which proportion of trials (e.g. from 2017, 2018 … 2021 or by study location) are registered, prospective registered and showed agreement between registry and publication. In my opinion, these are the very informative results here that are important to know for the reader. This is also true for totals, the important information is not that the crude denominator with agreements is 209 but that the proportion is 209/285=73.3%. Maybe you can also show both proportions (and maybe it would also be more appropriate not to show proportions with (dis)agreement here in table 3 but in table 5, see my comment below).

**Reply:** Thank you for your valuable feedback. We have revised Table 3 and optimized Table 5 to connect the information better and present the proportions of (dis)agreement more appropriately. We believe these changes will enhance the clarity and informativeness of our results.

**Changes in the text:** Please see the revised Tables 3 (above) and Table 5.

Table 5. Univariate analyses of factors related to a discrepancy of the primary outcome in the publication.

| **Characteristic** | **Total trial** | **Primary-Outcome agreement trial, N (%)** | **Primary-Outcome disagreement trial, N (%)** | **Univariate analyses** | |
| --- | --- | --- | --- | --- | --- |
|  | **N=285** | **N=209** | **N=76** | **Odds ratio (95% CI)** | **P-value** |
| **Quartile** | | | | | |
| Q4 ^a^ | 41 | 27(65.9) | 14(34.1) |  |  |
| Q3 | 36 | 27(75.0) | 9(25.0) | 0.576 (0.276,1.202) | 0.14 |
| Q2 | 34 | 21(61.8) | 13(38.2) | 1.194 (0.464,3.075) | 0.71 |
| Q1 | 174 | 134(77.0) | 40(23.0) | 0.643 (0.238,1.735) | 0.38 |
| **Year** | | | | | |
| 2017 ^a^ | 56 | 37(66.1) | 19(33.9) |  |  |
| 2018 | 50 | 39(78.0) | 11(22.0) | 0.998 (0.452,2.207) | >0.99 |
| 2019 | 68 | 47(69.1) | 21(30.9) | 0.548 (0.228,1.320) | 0.18 |
| 2020 | 58 | 51(87.9) | 7(12.1) | 0.869 (0.404,1.870) | 0.72 |
| 2021 | 53 | 35(66.0) | 18(34.0) | 0.267 (0.101,0.706) | 0.008* |
| **Country** | | | | | |
| Europe ^a^ | 120 | 85(70.8) | 35(29.2) |  |  |
| America | 82 | 65(79.3) | 17(20.7) | 0.824 (0.072,9.378) | 0.88 |
| Asia | 72 | 50(69.4) | 22(30.6) | 0.523(0.045,6.117) | 0.61 |
| Oceania | 8 | 7(87.5) | 1(12.5) | 0.880 (0.076,10.221) | 0.92 |
| Africa | 3 | 2(66.7) | 1(33.3) | 0.286 (0.012,6.914) | 0.44 |
| **Study center** | | | | | |
| Single center ^a^ | 74 | 51(68.9) | 23(31.1) |  |  |
| Multicenter | 177 | 133(75.1) | 44(24.9) | 1.088 (0.472,2.507) | 0.84 |
| Not specified | 34 | 25(73.5) | 9(26.5) | 1.363 (0.749,2.481) | 0.31 |
| **Phase of study** | | | | | |
| I ^a^ | 10 | 8(80) | 2(20) |  |  |
| II | 64 | 49(76.6) | 15(23.4) | 0.563 (1.114,2.768) | 0.48 |
| III | 54 | 42(77.8) | 12(22.2) | 0.689(0.346,1.137) | 0.29 |
| IV | 27 | 20(74.1) | 7(25.9) | 0.643 (0.306,1.350) | 0.24 |
| Not specified | 130 | 90(69.2) | 40(30.8) | 0.788 (0.308,2.012) | 0.62 |
| **Funding** | | | | | |
| Profit ^a^ | 94 | 76(80.9) | 18(19.1) |  |  |
| Non-profit | 128 | 88(68.8) | 40(31.2) | 0.533 (0.244,1.164) | 0.11 |
| Mixed | 11 | 9(81.8) | 2(18.2) | 1.023 (0.509,2.054) | 0.95 |
| None/NA | 52 | 36(69.2) | 16(30.8) | 0.500 (0.097,2.581) | 0.41 |
| **Trial design** | | | | | |
| Superiority ^a^ | 264 | 191(72.3) | 73(27.7) |  |  |
| Equivalency | 3 | 3(100.0) | 0(0) | 1.911 (0.537,6.795) | 0.32 |
| Noninferiority | 18 | 15(83.3) | 3(16.7) | 0 | >0.99 |
| **Registration status** | | | | | |
| Prospective ^a^ | 227 | 171(75.3) | 56(24.7) |  |  |
| Retrospective | 58 | 38(65.5) | 20(34.5) | 1.607 (0.865,2.987) | 0.13 |
| **CONSORT endorsed** | | | | | |
| No ^a^ | 238 | 172(72.3) | 66(27.7) |  |  |
| Yes | 47 | 37(78.7) | 10(21.3) | 1.420 (0.668,3.018) | 0.36 |
| **Mandatory RCT registration** | | | | | |
| Not ^a^ | 68 | 51(75) | 17(25) |  |  |
| Yes | 217 | 158(72.8) | 59(27.2) | 0.893(0.478,1.668) | 0.72 |

Q: Quartile; CONSORT: Consolidated Standards of Reporting Trials; RCT: randomized controlled trials; CI: confidence interval.

^a^: Reference category; ***: p< 0.05

**Comment 10**

Table 5 is also a difficult one. You show that in 2021 a statistically significant lower proportion of disagreements was found. But, again, the proportion of (dis)agreements by years (or any other covariates) is never shown. What you are showing here is not the information the reader needs. Overall, tables 3 and 5 need to be rearranged and more stratified proportions should also be described in the text.

**Reply:** Thank you for your insightful feedback. Similar to comment 9, we have revised Table 3 and optimized Table 5 to connect the information better and present the proportions of (dis)agreement more appropriately. We believe these changes will enhance the clarity and informativeness of our results.

**Changes in the text:** Please see the revised Tables 3 and Table 5 presented above.

**Comment 11**

Furthermore, in the header of table 5, odds ratios from the logistic regression should also be named as such (as you also showed kappa coefficients, which would otherwise be confusing for the reader).

**Reply:** We thank the reviewer for the suggestion. We have revised it.

**Changes in the text:** The term "Coefficient (95% CI)" has been revised to "Odds Ratio (95% CI)." in the header of table 5.

**Comment 12**

Discussion: A (although weak) reason for not registering a trial maybe that a study protocol was published beforehand. Did you assess this?

**Reply:** We appreciate your insight into this aspect. Our study did not assess the publication of study protocols beforehand. We recognize that this could be a relevant consideration and address it as a limitation in our manuscript.

**Changes in the text:** Lines 373-376 "Although we conducted a detailed registration search in platforms to confirm whether a study was registered or not, we did not evaluate whether study protocols were published prior to the registration of the trials. It is possible that some trials were not registered due to the existence of a prior published protocol. Future research could benefit from investigating this aspect".

**Comment 13**

Furthermore, to get it right, what about studies that did not report registration (or a registration number) but were indeed registered. Have you checked that possibility and how often was that the case? Maybe this also needs to be more elaborated in methods sections as it is not clear how you assessed whether a study was registered or not. Along with that, you write that you found invalid registration numbers (line 299). How did you deal with that (was this a non-registered study)?

**Reply:** Thank you for your insightful question. To ensure we did not miss any registration numbers, we adopted a thorough approach inspired by Killeen et al. and Rankin et al. Specifically, we undertook the following steps to identify trial registry numbers: 1. Verification through the WHO International Clinical Trials Registry Platform (ICTRP): This platform consolidates trial registrations from various registries worldwide, providing a comprehensive resource for verifying the validity of registry numbers. 2. Supplementary Searches: In cases where the registry number was not immediately apparent, we conducted additional searches based on the trial's publication title, authors' names, trial participants, and primary sponsors. This supplementary search aimed to cross-reference and uncover any potential registrations that might have been missed initially.

We deemed the trial unregistered if a trial's registry number could not be identified through these extensive efforts. This approach ensures that we are as exhaustive and accurate as possible in our identification process, thereby maintaining the reliability of our methodology.

If the authors provided a registration number but we did not find a corresponding registration record, we treated these trials in the same manner as studies that did not report a registration number at all. We found a total of 10 invalid registration numbers, and after thorough searches, we could not locate any corresponding registration records.

References:

1) Killeen S, Sourallous P, Hunter IA, Hartley JE, Grady HL. Registration rates, adequacy of registration, and a comparison of registered and published primary outcomes in randomized controlled trials published in surgery journals. Ann Surg. 2014 Jan;259(1):193-6.

2) Rankin J, Ross A, Baker J, O'Brien M, Scheckel C, Vassar M. Selective outcome reporting in obesity clinical trials: a cross-sectional review. Clin Obes. 2017 Aug;7(4):245-254.

**Changes in the text**: None.

**Comment 14**

One important piece of information would also be which endpoints were most often changed (e.g. mortality or quality of life) and in which studies (e.g. on drugs effectiveness or screening). Was this assessed or is there some kind of data from the literature on that?

**Reply:** Thank you for your insightful comments. The primary aim of this study is to examine the overall discrepancies and understand the dimensions of these discrepancies, as outlined in the five categories listed in Table 4. While your suggestion regarding the specific endpoints (e.g., mortality or quality of life) and their changes in various types of studies (e.g., drug effectiveness or screening) is valuable, it is not the primary concern of this article. Upon revisiting the 76 articles with discrepancies, we found that the scope of these trials is quite broad, making it challenging to categorize endpoints based on specific content dimensions such as mortality or quality of life. However, we have added a sentence in the discussion to address this point: "Nevertheless, whether certain endpoints are more prone to discrepancies was not the focus of this study and warrants further investigation in future research."

**Changes in the text:** Lines 334-335 "In addition, whether certain endpoints are more prone to discrepancies was not the focus of this study and warrants further investigation in future research."

**Comment 15**

On line 310-313, you discuss that your findings of higher proportions of disagreements than in earlier studies might imply that a divergence has concurrently risen. However, this is a bad argument not supported by your data, where in 2021 this proportions was even lower. Are there any other methodological explanations?

**Reply:** Thank you for your comment. We would like to clarify that our findings indicate a 26.7% (76/285) proportion of discrepancies compared to 14.2% in the earlier study. Therefore, our data does support the argument that there may be a concurrent rise in outcome reporting bias.

**Changes in the text:** None.

**Comment 16**

Furthermore, there might be (more or less plausible) reasons to change endpoints, that, of course, need to be transparently discussed in a study publication. Have you assessed how often this was the case (as you write in line 320 that in many cases this was not explained)? When you have assessed this systematically, these data need to be presented. Otherwise, you need to be somewhat more cautious in your own discussion.

**Reply:** Thank you for your insightful comments and suggestions. Following your suggestion, we systematically reviewed the 76 papers in our study that exhibited changes to primary endpoints. Our assessment revealed that none of them provided an explanation for the changes made. However, we did find instances where studies described reasons for changes in study protocols. For example, one study modified its protocol to include a CT scan at 3 weeks post-intervention due to adverse events observed at that time, instead of the originally planned 6-week post-intervention assessment (<https://www.ncbi.nlm.nih.gov/pmc/articles/PMC6582745/>). This change was recommended by the safety committee to better monitor treatment response and patient safety.

We have revised the discussion section to incorporate these findings and provide a more nuanced understanding of the discrepancies in primary outcome reporting.

**Changes in the text:** Lines 325-335 “…Additionally, omitting and introducing a primary outcome were the second and third most common discrepancies. A point claimed that the discrepancies were caused by the researchers’ discovery of unintended effects or harms caused by the intervention, leading to the selective omission of pre-specified primary outcomes [29]. However, it is important to note that changes to primary endpoints do not inherently indicate poor practice. There can be plausible reasons for such changes, which should be transparently discussed in the study publication. Our review of the 76 papers with primary endpoint changes revealed that none provided explanations for the changes to the primary outcomes. The lack of explanations for primary outcome changes does not necessarily imply issues with the study but highlights a gap in procedural transparency. Without these explanations, assessing the appropriateness and rationale behind the changes is challenging. Therefore, while our findings suggest a higher rate of discrepancies in primary outcome reporting, it is crucial to approach these results with caution. In addition, whether certain endpoints are more prone to discrepancies was not the focus of this study and warrants further investigation in future research.”

**Comment 17**

I do not understand what you mean with the inabilities in the registry (lines 357-359).

**Reply:** We apologize for the confusion. We realize that our previous description may have been unclear. While the ICTRP provides a comprehensive registry, it does not always allow for a clear assessment of historical versions or the exact trial stage at which changes were made. For instance, prospective registrations may have retrospective changes where authors modify primary outcomes after concluding the trial. Therefore, we compared the primary outcomes specified at the time of the initial registration and did not include any outcomes added or modified in subsequent updates. We have decided to remove this point from the manuscript as it is not a limitation of our study.

**Changes in the text:** We have deleted the sentence "the inability to discern updates, revisions, or supplements to primary outcomes in the International Clinical Trials Registry Platform may limit the accuracy of primary outcome extraction, as only initially registered primary outcomes were considered".

**Comment 18**

Other limitations include the restrictions of your work to one field and a small period of time.

**Reply:** Many thanks for your suggestion. We have added this limitation.

**Changes in the text:** Lines 370-373 "Thirdly, this study is restricted to a specific field and covers a relatively small period of time, specifically five years. Consequently, the findings of this study may not be generalizable to other fields or longer timeframes. Further research is needed to explore these aspects in different contexts and over extended periods".

**Comment 19**

In my opinion, implications and actions needed (lines 361-382) are some kind of superficial but at the same time also too enthusiastic. Is there really a need for a cultural shift (this is a very strong statement)? Is there evidence that more and more journals recognize non-significant results (and since when is this the case)?

**Reply:** Thank you for your insightful comments. We want to address your concerns about the necessity of a cultural shift and the evidence supporting journals’ increasing recognition of non-significant results. We believe that a cultural shift is indeed necessary within the scientific community to embrace and value negative or non-significant findings. Research indicates that the bias towards publishing statistically significant results has been a longstanding issue. Sterling's 1959 study highlighted that studies with significant findings were more likely to be published than those with non-significant results (1). Subsequently, more authors found the finding of statistically significant outcomes having a higher likelihood of getting published than null results has been confirmed (2-4).

Meanwhile, evidence also shows a gradual shift. A study covering publication trends from 1985 to 2013 found that while significant results still dominate, there is a notable increase in the reporting of non-significant results across various journals, suggesting a slow but positive change in attitudes towards these findings (5). Thank you once again for your valuable feedback.

References:

(1) Sterling T D. Publication decisions and their possible effects on inferences drawn from tests of significance—or vice versa. Journal of the American Statistical Association. 1959; 54; 30–34. 10.1080/01621459.1959.10501497

(2) Franco A, Malhotra N, Simonovits G. Publication bias in the social sciences: Unlocking the file drawer. Science. 2014; 345; 1502–1505. doi: 10.1126/science.1255484

(3) Koren G, Fernandes A. Reviewers’ bias against the null hypothesis: The reproductive hazards of binge drinking. Journal of Population Therapeutics and Clinical Pharmacology. 2010; 17; 281–283.

(4) Hopewell S, Loudon K, Clarke M J, Oxman A D, Dickersin K. Publication bias in clinical trials due to statistical significance or direction of trial results. Cochrane Database of Systematic Reviews. 2009; 1; 1–26. doi: 10.1002/14651858.MR000006.pub3

(5) Hartgerink, C. H. J., et al. (2017). Too Good to be False: Nonsignificant Results Revisited. Collabra: Psychology, 3(1): 9. doi: https://doi.org/10.1525/collabra.71

**Changes in the text:** None.

**Comment 20**

Is there really a need for training of reviewers (lines 369-372)? Is it the task of a reviewer to check whether endpoints were changed? In the following paragraph you write, that this should be done by the journal.

**Reply:** We appreciate your concerns. We intended to emphasize the importance of collaboration between reviewers and editors in ensuring the consistency between registered and reported primary outcomes. While it may not be solely the task of a reviewer to check for endpoint changes, enhanced training can enable reviewers to provide more comprehensive and professional feedback. We have made additions to address the comment.

**Changes in the text:** Lines 392-396 "Thirdly, the observed shortcomings in the quality of RCT reviews suggest that raising the awareness among editors and reviewers involved in assessing RCTs to check registration information is crucial. Editors could receive training to strengthen their capabilities to rigorously evaluate trial protocols, registration details, and outcome reporting. One underlying issue is inadequate training sources and content, a key issue which requires attention [33]. Reviewers should work closely with editors to ensure consistency between registration and reporting in manuscripts, and make more specialized comments when needed."

**Comment 21**

What about authors? They need to report such changes transparently. This should be trained in courses on good clinical practice and biometricians or clinical study centers need also be aware of this (as they often supervising many RCTs).

**Reply:** We appreciate your feedback. We agree that authors play a crucial role in ensuring the transparency of reporting changes and minimizing reporting bias. It is essential to train authors in good clinical practice. Additionally, it is important to extend this training to other key participants in clinical trials, such as biometricians and clinical study centers, who often supervise multiple RCTs. We revised the implications and actions section to include this important point.

**Changes in the text:** Lines 386-390 "Secondly, it is imperative to provide comprehensive training for authors. For instance, emphasizing the importance of trial registration, updating registries when primary outcomes are modified, and transparently reporting both positive and negative results. Moreover, training should extend beyond authors to include biostatisticians, clinical research coordinators, and staff at clinical study centers. This comprehensive training approach ensures that all parties involved in clinical trials are aware of the standards necessary for transparent reporting".
