# Supplementary material for: Discrepancies between pre-specified and reported primary outcomes: A cross-sectional analysis of randomized controlled trials in gastroenterology and hepatology journals
Source: PLoS One. 2024 Nov 22;19(11):e0305027. doi: 10.1371/journal.pone.0305027 (PMC11584078; doi:10.1371/journal.pone.0305027)
Supplement: Attachment — Submitted filename: PONE-S-24-26486-R2-Response to reviewers.docx [file pone.0305027.s006.docx]

**ID:** **PONE-S-24-26486**

**Manuscript:** **Discrepancies between pre-specified and reported primary outcomes: a cross-sectional analysis of randomized controlled trials in gastroenterology and hepatology journals**

Dear Academic Editor Sascha Köpke, Dr. Martin Holst (Reviewer #1) and Reviewer #2,

We are very grateful to you for the constructive comments on our paper, which have substantially improved the overall quality of this manuscript. All the comments have been carefully reviewed and addressed, with corresponding changes denoted in the manuscript. A clean, unannotated copy of the revised paper is also provided for your review. Additionally, we have provided further explanations where needed. We believe these revisions have strengthened the manuscript and made it more informative for our readers. Once again, thank you for your invaluable input. We look forward to your feedback on the revised manuscript.

Best Regards,

Kaiping Zhang, PhD, MPH

**Point-to-point Reply to Reviewers’ Comments**

**Reviewer #1:**

Dear authors, dear editor,

thank you for the opportunity to again review the manuscript entitled “Discrepancies between pre-specified and reported primary outcomes: a cross-sectional analysis of randomized controlled trials in gastroenterology and hepatology journals.”

I would like to thank the authors for their extensive efforts to implement the changes requested by the reviewers, including reworking all the tables (which I find much more readable now!). I believe the manuscript has greatly improved and can be accepted for publication. There are, however, three minor points I would like to address:

**Reply:** Thank you for your positive feedback and efforts put in reviewing our manuscript.

**Comment 1**

(1) My Comment 6
You provided a detailed answer to my question, thank you! However, you made no changes to the text. I would appreciate if you could explain this to readers, too.
**Reply:** Many thanks for the suggestion. We have added this in the revised manuscript.

**Changes in the text:** Lines 175-179 “…or if a nonsignificant primary outcome was omitted (e.g., the omitted outcome might not have achieved statistical significance, leading to its exclusion from the published results. This assumption is based on the notion that statistically significant results are more likely to be reported and published due to the well-known publication bias) or defined as nonprimary in the published articles, or if registered statistically significant secondary outcomes became published primary outcomes.”

**Comment 2**

(2) My Comment 16

You added that there was only one paper for which you could not obtain the full text. Could you also add this information to the manuscript?
**Reply:** Thank you for bringing this to our attention. We have revised it.

**Changes in the text:** Lines 226-228 “Subsequently, 405 articles underwent full-text screening for eligibility. Of them, 42 cross-over studies and ancillary studies were further excluded. And we were unable to obtain the full text of one trial. Finally, 362 trials were eligible for inclusion.”

**Comment 3**

(3) My Comment 18

Thank you for providing a detailed explanation! I think this was a misunderstanding, however. I had just been wondering - should the sentence not read: “If NO registry number was uncovered in this way, the trial was deemed unregistered.” If this is a misunderstanding from my side, please feel free to ignore the comment.
**Reply:** We sincerely appreciate your informative comment and apologize for this misunderstanding writing. We now have changed it.

**Changes in the text:** Lines 151-152 “If no registry number was uncovered in this way, the trial was deemed unregistered.”

**Reviewer #2:**

Thank you for your valuable response and for revising the manuscript. Most of my points are addressed, but there are still some things that should be improved.

**Reply:** Thank you for your positive feedback and efforts put in reviewing our manuscript.

**Comment 1**

Introduction: On line 101, it still remains unclear why a study from the field of gastroenterology is needed and why results should differ from other fields.
**Reply:** Thank you for your valuable feedback. We apologize for any confusion caused by our previous submission. Regarding your first concern, the burden of gastrointestinal diseases is substantial, accounting for more than one-third of prevalent disease cases globally in 2019. Despite this significant burden, research exploring the consistency between registered and published primary outcomes in the field of gastroenterology remains limited. To our knowledge, only one relevant study by Li et al. has addressed this issue within high-impact gastroenterology and hepatology journals. This scarcity of research underscores the necessity of our study to fill this gap.

Regarding your second concern, we have also reviewed literature from other biomedical fields, which has reported discrepancies between registered and published primary outcomes ranging from 5% to 92%. These variations are likely influenced not only by differences in the study design, but also by various of biomedical fields. We hope that our additional explanations and the context provided address your concerns and clarify the necessity of our study in this field.

**Changes in the text:** Lines 74-83 “Gastroenterology and hepatology are fields that often involve multifaceted treatment regimens and diverse patient populations. In 2019, digestive diseases accounted for more than one-third of prevalent disease cases, representing a significant global health care burden [1]. In the era of evidence-based medicine, high-quality randomized controlled trials (RCTs) stand as pivotal sources of evidence in scientific research, owing to their robust study designs and significant value [2]. These trials often serve as primary references for formulating clinical guidelines and shaping medical decision-making. However, numerous trials encounter the issue of selective and incomplete reporting of results, which distorts their evidence-based value [3-6]. The Centre for Evidence-Based Medicine Outcome Monitoring Project has found that, on average, each trial in top-ranked medical journals silently adds 5.3 new outcomes [7]. Such discrepancies in trial results can potentially exaggerate benefits or underestimate adverse outcomes, leading to misguided clinical recommendations, wastage of resources, and, in severe cases, harm to patients [8].”

Lines 95-99, “Moreover, selective outcome reporting bias persists across biomedical disciplines, including anesthesiology, psychology, otorhinolaryngology, headache medicine, plastic surgery, mental health and orthopaedical surgery despite the implementation of ICMJE guidelines. Studies in these biomedical fields have reported a huge field difference in discrepancies between registered and published primary outcomes, ranging from 5% to 92% [13-19].”

Line 101, “Despite the huge field difference, research on this important topic remains limited in the field of gastroenterology and hepatology.”

References:

1. Wang Y, Huang Y, Chase RC, Li T, Ramai D, Li S, et al. Global Burden of Digestive Diseases: A Systematic Analysis of the Global Burden of Diseases Study, 1990 to 2019. Gastroenterology. 2023;165(3):773-83 e15. [http://doi.org/10.1053/j.gastro.2023.05.050. PMID:37302558](http://doi.org/10.1053/j.gastro.2023.05.050.%20PMID:37302558).

18. Vrljicak Davidovic N, Komic L, Mesin I, Kotarac M, Okmazic D, Franic T. Registry versus publication: discrepancy of primary outcomes and possible outcome reporting bias in child and adolescent mental health. Eur Child Adolesc Psychiatry. 2022;31(5):757-69. http://doi.org/10.1007/s00787-020-01710-5. PMID:33459886.

19. Rongen JJ, Hannink G. Comparison of Registered and Published Primary Outcomes in Randomized Controlled Trials of Orthopaedic Surgical Interventions. J Bone Joint Surg Am. 2016;98(5):403-9. http://doi.org/10.2106/JBJS.15.00400. PMID:26935463.

**Comment 2**

On lines 105 and 107, there is now a redundancy on studies that did not assess discrepancies on journal quartiles and high vs. low impact journals.

**Reply:** Thank you for your comment. We have deleted the redundant sentence.

**Changes in the text:** Line 111 deleted the sentence “no recent study has examined the prevalence and nature of major outcome discrepancies in high-impact and lower-impact journals within this domain”.

**Comment 3**

Regarding my earlier comment 13, you still did not make clear how you proceed regarding whether a study was registered or not. This is important because different scenarios are possible (e.g. no registration number is in the publication and you a) did not find or b) did find a registration or c) the registration number is wrong). Please describe your approach in the methods section in more detail.

**Reply:** Thank you for your insightful comment. We apologize for any confusion. To clarify our approach in determining whether a study was registered, we adhered to the following detailed steps:

1) If no registration number was provided in the publication:

We manually searched the ICTRP using the publication title, author names, trial participants, and primary sponsors to identify any possible registration numbers. If no registry number was uncovered in this way, the trial was deemed unregistered.

2) If a registration number was provided in the publication:

We entered the registration number into the ICTRP to retrieve relevant registration information. If the authors provided a registration number but we did not find a corresponding registration record, we treated these trials in the same manner as studies that did not report a registration number at all.

This comprehensive approach ensures that we thoroughly verify the registration status of each study. We hope this clarifies our methodology.

**Changes in the text:** Lines 149-154 “…This approach ensured uniformity in search mechanisms. If no registration number was provided in the publication, we manually searched the ICTRP using the publication title, author names, trial participants, and primary sponsors to identify any possible registration numbers. If no registry number was uncovered in this way, the trial was deemed unregistered. If a registration number was provided in the publication, we entered the registration number into the ICTRP to retrieve relevant registration information. If the authors provided a registration number but we did not find a corresponding registration record, we treated these trials in the same manner as studies that did not report a registration number at all.”

**Comment 4**

On line 197, it does not make sense that a binary regression included all significant variables in the univariate analysis, as both approaches assess the same and all variables were included.
**Reply:** Thank you for pointing out the issue. We apologize for the oversight and any confusion it may have caused. This was indeed a typographical error on our part. We have changed “binary logistic regression” to “multivariable logistic regression”. To clarify, our actual approach was to first conduct univariate analysis. Only variables that showed significant differences in the univariate analysis were then included in the multivariable logistic regression analysis. As indicated by the results in Table 5, only one independent variable was significant. Therefore, we did not proceed with multivariable regression analysis. And we also add this point in the Results section “Only the publication year was associated with differences in primary outcome reporting; therefore, multivariable logistic regression analysis was not conducted”. We appreciate your attention to detail and hope this explanation resolves the confusion.

**Changes in the text:** Lines 194-197 “Additionally, univariate analyses are performed to determine the effect of each variable on primary outcome discrepancies. Multivariable logistic regression includes all the significant variables in the univariable analysis to identify factors influencing discrepancies between the registered and reported primary outcomes.

Lines 266-270 “Univariate analyses revealed that only the publication year (2020) was associated with the discrepancy between registered and published primary outcomes (OR=0.267, 95%CI:0.101,0.706, p=0.008) (Table 5); therefore, multivariable logistic regression analysis was not conducted.”

**Comment 5**

Table 3c is the same as table 5, which is confusing. Please delete table 3c.
**Reply:** Thank you for your thoughtful comment and we have deleted Table 3c.

**Changes in the text:** Please see revised Table 3.

Table 3. Registration and prospective registration across clinical trials, stratified by trial characteristics.

| **Table 3a** | | | | |
| --- | --- | --- | --- | --- |
| **Feature** | **Total (n=362)** | **Registered trials (n=312)** | **Unregistered trials(n=50)** | **P-Value** |
| **Year of publication, n (%)** |  |  |  | 0.83 |
| 2017 | 73 | 63(86.3) | 10(13.7) |  |
| 2018 | 64 | 52(81.3) | 12(18.7) |  |
| 2019 | 82 | 76(92.7) | 6(7.3) |  |
| 2020 | 75 | 61(81.3) | 14(18.7) |  |
| 2021 | 68 | 60(88.2) | 8(11.8) |  |
| **Study Location, n (%)** |  |  |  |  |
| Europe | 148 | 131(88.5) | 17(11.5) | 0.009 * |
| America | 100 | 91(91.0) | 9(9.0) |  |
| Asia | 99 | 78(78.8) | 21(21.2) |  |
| Oceania | 9 | 9(100.0) | 0(0.0) |  |
| Africa | 6 | 3(50.0) | 3(50.0) |  |
| **Quartile, n (%)** |  |  |  | <0.001* |
| Q1 | 197 | 185(93.9) | 12(6.1) |  |
| Q2 | 52 | 39(75.0) | 13(25.0) |  |
| Q3 | 50 | 39(78.0) | 11(22.0) |  |
| Q4 | 63 | 49(77.8) | 14(22.2) |  |
| **Funding, n (%)** |  |  |  | 0.01* |
| Industry | 109 | 97(89.0) | 12(11.0) |  |
| Nonprofit | 156 | 139(89.1) | 17(10.9) |  |
| Mixed | 17 | 16(94.1) | 1(5.9) |  |
| None disclosed/no funding | 80 | 60(75.0) | 20(25.0) |  |
| **Trial design, n (%)** |  |  |  | 0.21 |
| Superiority | 338 | 289(85.5) | 49(14.5) |  |
| Equivalency | 4 | 4(100.0) | 0(0.0) |  |
| Noninferiority | 20 | 19(95.0) | 1(5.0) |  |

| **Table 3b** | | | | |
| --- | --- | --- | --- | --- |
| **Feature** | **Total (n=312)** | **Prospective Registration (n=249)** | **Retrospective Registration (n=63)** | **P-value** |
| **Year of publication, n (%)** |  |  |  | 0.92 |
| 2017 | 63 | 51(81.0) | 12(19.0) |  |
| 2018 | 52 | 42(80.8) | 10(19.2) |  |
| 2019 | 76 | 57(75.0) | 19(25.0) |  |
| 2020 | 61 | 53(86.9) | 8(13.1) |  |
| 2021 | 60 | 46(76.7) | 14(23.3) |  |
| **Study Location, n (%)** |  |  |  | 0.74 |
| Europe | 131 | 105(80.2) | 26(19.8) |  |
| America | 91 | 76(83.5) | 15(16.5) |  |
| Asia | 78 | 59(75.6) | 19(24.4) |  |
| Oceania | 9 | 7(77.8) | 2(22.2) |  |
| Africa | 3 | 2(66.7) | 1(33.3) |  |
| **Quartile, n (%)** |  |  |  | <0.001* |
| Q1 | 185 | 163(88.1) | 22(11.9) |  |
| Q2 | 39 | 25(64.1) | 14(35.9) |  |
| Q3 | 39 | 31(79.5) | 8(20.5) |  |
| Q4 | 49 | 30(61.2) | 19(38.8) |  |
| **Funding, n (%)** |  |  |  | 0.001* |
| Industry | 97 | 90(92.8) | 7(7.2) |  |
| Nonprofit | 139 | 102(73.4) | 37(26.6) |  |
| Mixed | 16 | 14(87.5) | 2(12.5) |  |
| None disclosed/no funding | 60 | 43(71.7) | 17(28.3) |  |
| **Trial design, n (%)** |  |  |  | 0.006* |
| Superiority | 289 | 230(79.6) | 59(20.4) |  |
| Equivalency | 4 | 1(25.0) | 3(75.0) |  |
| Noninferiority | 19 | 18(94.7) | 1(5.3) |  |

Q: Quartile.

*: P< 0.05.

**Comment 6**

What is the outcome for the odds ratios in table 5, agreement or disagreement?

**Comment 7**

Regarding the year in table 5 and in the text you write that 2021 was significant different (OR=0.267). However, the proportions with disagreements between 2017 and 2021 are the same (33.9% and 34.0%). Something is wrong here. Maybe this result is for 2020. Furthermore, the odds ratios for 2018 and 2019 also do not fit together.

**Comment 8**

The same seem to hold true for other analyses in table 5, e.g. the proportion from Europe and Asia are quite the same (29.2% and 30.6%) but the odds ratio is given to be 0.523. Please check all analyses carefully.
**Reply:** Thank you for your insightful comments regarding Table 5. Your comments prompted a thorough review of our analyses, leading to important corrections. Below, we address your comments in detail:

Regarding to comment 6, the odds ratios (OR) presented in Table 5 pertain to the likelihood of disagreement between registered and published primary outcomes. This means that the ORs reflect the relationship between various independent variables (e.g., publication year, quartile) and the probability of disagreement, which can indicate either protective or risk factors. An OR greater than 1 suggests a higher likelihood of discrepancy (risk factor), while an OR less than 1 suggests a lower likelihood of discrepancy (protective factor).

Regarding to comment 7, you are correct; there was an error in the initial analysis. After re-evaluating our univariate analyses, we realized that the mistakes were due to an incorrect definition of reference category, which led to inconsistencies between the OR values and the reported proportions. For example, in the revised Table 5, the OR=0.267, 95% CI (0.101, 0.706), p=0.008 indicates that the likelihood of primary outcome discrepancy in 2020 is 0.267 times that of 2021, and this difference is statistically significant. The proportion of studies with primary outcome discrepancies in 2020 was lower compared to 2021 (12.1% vs 34%).

As for the study location, we corrected the reference variable to Africa. After this correction, the ORs now correctly reflect the data.

Overall, we appreciate your insights, which have led us to refine our analyses. We believe these corrections address the issues you raised and enhance the clarity of our findings.

**Changes in the text:** Please see the revised Table 5, lines 47-48 in the Abstract “Univariate analyses revealed that only the publication year (year 2020) was associated with the discrepancy between registered and published primary outcomes (OR=0.267, 95% CI: 0.101, 0.706, p=0.008)”, and lines 265-272 “Univariate analyses revealed that only the publication year (year 2020) was associated with the discrepancy between registered and published primary outcomes (OR=0.267, 95%CI:0.101,0.706, p=0.008) (Table 5); therefore, multivariable logistic regression analysis was not conducted. Specifically, the result indicated that in 2020, the likelihood of primary outcome discrepancy was 0.267 times that of 2021, and this difference was statistically significant. The proportion of studies with primary outcome discrepancies was lower in 2020 compared to 2021 (12.1% vs 34%). Journal quartile, study location, study center, phase of the study, funding, trial design, registration status, CONSORT endorsed, and whether RCT registration was mandatory were not associated with the discrepancy of registered and published primary outcomes.”

Table 5. Univariate analyses of factors related to a discrepancy of the primary outcome in the publication.

| **Characteristic** | **Total, N** | **Primary-Outcome agreement trial, N (%)** | **Primary-Outcome disagreement trial, N (%)** | **Univariate analyses** | |
| --- | --- | --- | --- | --- | --- |
|  | **N=285** | **N=209** | **N=76** | **Odds ratio (95% CI)** | **P-value** |
| **Quartile** | | | | | |
| Q4 ^a^ | 41 | 27(65.9) | 14(34.1) |  |  |
| Q1 | 174 | 134(77.0) | 40(23.0) | 0.576 (0.276,1.202) | 0.14 |
| Q2 | 34 | 21(61.8) | 13(38.2) | 1.194 (0.464,3.075) | 0.71 |
| Q3 | 36 | 27(75.0) | 9(25.0) | 0.643 (0.238,1.735) | 0.38 |
| **Publication year** | | | | | |
| 2021 ^a^ | 53 | 35(66.0) | 18(34.0) |  |  |
| 2017 | 56 | 37(66.1) | 19(33.9) | 0.998 (0.452,2.207) | >0.99 |
| 2018 | 50 | 39(78.0) | 11(22.0) | 0.548 (0.228,1.320) | 0.18 |
| 2019 | 68 | 47(69.1) | 21(30.9) | 0.869 (0.404,1.870) | 0.72 |
| 2020 | 58 | 51(87.9) | 7(12.1) | 0.267 (0.101,0.706) | 0.008* |
| **Study location** | | | | | |
| Africa ^a^ | 3 | 2(66.7) | 1(33.3) |  |  |
| Europe | 120 | 85(70.8) | 35(29.2) | 0.824 (0.072,9.378) | 0.88 |
| America | 82 | 65(79.3) | 17(20.7) | 0.523(0.045,6.117) | 0.61 |
| Asia | 72 | 50(69.4) | 22(30.6) | 0.880 (0.076,10.221) | 0.92 |
| Oceania | 8 | 7(87.5) | 1(12.5) | 0.286 (0.012,6.914) | 0.44 |
| **Study center** | | | | | |
| Multicenter ^a^ | 177 | 133(75.1) | 44(24.9) |  |  |
| Single center | 74 | 51(68.9) | 23(31.1) | 1.088 (0.472,2.507) | 0.84 |
| Not specified | 34 | 25(73.5) | 9(26.5) | 1.363 (0.749,2.481) | 0.31 |
| **Phase of study** | | | | | |
| Not specified ^a^ | 130 | 90(69.2) | 40(30.8) |  |  |
| I | 10 | 8(80) | 2(20.0) | 0.563 (1.114,2.768) | 0.48 |
| II | 64 | 49(76.6) | 15(23.4) | 0.689(0.346,1.137) | 0.29 |
| III | 54 | 42(77.8) | 12(22.2) | 0.643 (0.306,1.350) | 0.24 |
| IV | 27 | 20(74.1) | 7(25.9) | 0.788 (0.308,2.012) | 0.62 |
| **Funding** | | | | | |
| Mixed ^a^ | 11 | 9(81.8) | 2(18.2) |  |  |
| None/NA | 52 | 36(69.2) | 16(30.8) | 2.000 (0.387,10.325) | 0.408 |
| Profit | 94 | 76(80.9) | 18(19.1) | 1.066 (0.212,5.364) | 0.938 |
| Non-profit | 128 | 88(68.8) | 40(31.2) | 2.045 (0.423,9.902) | 0.374 |
| **Trial design** | | | | | |
| Noninferiority ^a^ | 18 | 15(83.3) | 3(16.7) |  |  |
| Superiority | 264 | 191(72.3) | 73(27.7) | 1.911 (0.537,6.795) | 0.32 |
| Equivalency | 3 | 3(100.0) | 0(0) | 0 | >0.99 |
| **Registration status** | | | | | |
| Prospective ^a^ | 227 | 171(75.3) | 56(24.7) |  |  |
| Retrospective | 58 | 38(65.5) | 20(34.5) | 1.607 (0.865,2.987) | 0.13 |
| **CONSORT endorsed** | | | | | |
| Yes ^a^ | 47 | 37(78.7) | 10(21.3) |  |  |
| No | 238 | 172(72.3) | 66(27.7) | 1.420 (0.668,3.018) | 0.36 |
| **Mandatory RCT registration** | | | | | |
| Yes ^a^ | 217 | 158(72.8) | 59(27.2) |  |  |
| Not | 68 | 51(75) | 17(25.0) | 0.893(0.478,1.668) | 0.72 |

Q: Quartile; CONSORT: Consolidated Standards of Reporting Trials; RCT: randomized controlled trials; CI: confidence interval.

^a^: Reference category; ***: P< 0.05

**Comment 9**

Regarding my comment 19, it would be nice when the arguments and references given could be discussed in the manuscript.

**Reply:** Many thanks for your suggestion. We have added this in the Discussion section.

**Changes in the text:** Lines 387-394, “Firstly, the prevalence of discrepancies within gastroenterology and hepatology journals, particularly those favoring statistically significant primary outcomes, underscores a broader issue within the scientific community—a prevailing bias against negative or non-significant results. Therefore, there is a pressing need for a cultural shift that embraces and values negative findings. Sterling highlighted in 1959 that studies with significant findings were more likely to be published than those with non-significant results [38]. Meanwhile, evidence also shows a gradual shift. A study in 2017 covering publication trends from 1985 to 2013 found that while significant results still dominate, there is a notable increase in the reporting of non-significant results across various journals, suggesting a slow but positive change in attitudes towards these findings [39]. This proactive approach would probably contribute to mitigating reporting bias.”

References:

38. Sterling TD. Publication decisions and their possible effects on inferences drawn from tests of significance—or vice versa. Journal of the American Statistical Association. 1959;54:30-4. http://doi.org/10.2307/2282137.

39. Hartgerink CHJ, Wicherts JM, van Assen MALM. Too Good to be False: Nonsignificant Results Revisited. Collabra: Psychology. 2017;3(1). http://doi.org/10.1525/collabra.71.
