# Supplementary material for: Discrepancies between pre-specified and reported primary outcomes: A cross-sectional analysis of randomized controlled trials in gastroenterology and hepatology journals
Source: PLoS One. 2024 Nov 22;19(11):e0305027. doi: 10.1371/journal.pone.0305027 (PMC11584078; doi:10.1371/journal.pone.0305027)
Supplement: Attachment — Submitted filename: PONE-D-20434-R3-Rebuttal letter 11.7.docx [file pone.0305027.s007.docx]

**ID:** **PONE-D-24-20434**

**Manuscript:** **Discrepancies between pre-specified and reported primary outcomes: a cross-sectional analysis of randomized controlled trials in gastroenterology and hepatology journals**

**Reviewer #2:**

Thank you for clarifying and improving the manuscript. There are few points regarding the differences for 2020 and 2021.

1. In the abstract, in 48 please describe that the OR for the publication year 2020 is in comparison to 2021.

2. In the discussion, please correct (line 298) “A more recent publication year (year 2021) was associated with a lower chance of primary outcome discrepancy.” This result now refers to 2020 and might not be explained by some kind of trend but rather by chance.

3. This also needs to be corrected in line 345 “Moreover, most baseline characteristics showed no significant influence on changes in primary outcomes, except for the year of publication, where more recent studies were associated with fewer outcome discrepancies.”

**Reply:** Thank you for your further careful check and bring these inconsistencies to us. We apologize the inconsistencies due to our missed inspections after correcting the reference group. We now have revised the mentioned places and checked through the manuscript.

**Changes in the text:**

1. Abstract, Lines47-48 “Univariate analyses revealed that primary outcome discrepancies were lower in the publication year 2020 compared to year 2021 (OR=0.267, 95% CI: 0.101, 0.706, p=0.008).”

2. Discussion, Lines 298-299 “Primary outcome discrepancies were lower in the publication year 2020 compared to year 2021 (OR=0.267, 95% CI: 0.101, 0.706, p=0.008).”

3. Discussion, Lines 345-346 “Moreover, most baseline characteristics showed no significant influence on changes in primary outcomes, except for the year of publication (year 2020 compared to 2021).”

4. Other revisions after checking through the manuscript are listed as below.

--Line 40, The word “binary logistic regression” is now corrected as “logistic regression”.

--Lines 46-47 “introducing new unregistered primary outcomes” is now corrected as “reporting the registered secondary outcomes as primary outcomes”.

--Lines 49-50, “20 studies were retrospectively registered and 32 (57.1%) showed statistically significant results” is now specified as “20 (26.3%) studies were retrospectively registered, and 32 (57.1%) of the prospectively registered trials with primary outcome discrepancies showed statistically significant results.”

--Lines 98-100, “headache medicine, plastic surgery, mental health and orthopaedical surgery despite the implementation of ICMJE guidelines…. ranging from 5% to 92% [13-19]” is now corrected as “headache medicine, mental health and orthopaedical surgery despite the implementation of ICMJE guidelines… ranging from 25.9% to 92% [13-18]”.

--Line 108, “Our article addresses this gap by focusing on journals ranked in the top three of each impact factor quartile in the JCR.”, the sentence is now deleted as this information can be found in the methods.

--Table 1, the subitem of “ICMJE member” is now corrected as “ICMJE member or adherence to ICMJE guidelines”. Therefore, on Line 210, “Notably, four journals, including Liver International, Colorectal Disease, Techniques in Coloproctology, and Journal of Pediatric Gastroenterology and Nutrition, were not members of the ICMJE (Table 1)”, the sentence is now deleted. Meanwhile, all the journals regarding ICMJE member or adherence to ICMJE guidelines in table 1 are now marked as “Yes”.

--Line 137, “Endnote” is now corrected as “Endnote (version 20; Clarivate Analytics, USA)”.

--Line 142, “Microsoft Excel” is now corrected as “Microsoft Excel (2022 version; Microsoft Corporation, USA)”.

--Line 182, “In cases where primary outcomes were added or changed, only the initially registered ones were extracted”, the sentence is now deleted as this information can be found above.

--Line 193, “The chi-square test or Fisher's exact test is used to assess differences in each trial characteristic across domains, including registration status, registration type, and primary outcome consistency” is now corrected as “The chi-square test or Fisher's exact test is used to assess differences in each trial characteristic across domains, including registration status and registration type”.

--Lines 199-200, “<0.2 indicating poor agreement” is now corrected as “≤0.2 indicating poor agreement”. “>0.81 very good agreement” is now corrected as “≥0.81 very good agreement”.

--Line 229, the sentence “while there was no difference in the number of RCTs published in Quartiles 2 to 4” is now deleted.

--Line 261, “introducing new unregistered primary outcomes” is now corrected as “registered secondary outcomes promoted to primary outcomes”.

--Line 262, “registered secondary outcomes promoted to primary outcomes in publications” is now corrected as “new primary outcomes introduced”.

--Line291, “with 68.8% registered predominantly prospectively” is now corrected as “with 86.2% (312 out of 362) registered and 79.8% (249 out of 312) registered predominantly prospectively”.

--Line 295, “introducing new unregistered primary outcomes” is now corrected as “reporting the registered secondary outcomes as primary outcomes”.

--Line 305, “with studies in psychiatry, pediatric surgery, anaesthesia, and urology reporting a range from 28.0% to 88.4% [14, 23-25]” is now corrected as “with studies in psychiatry, pediatric surgery, and anaesthesia reporting a range from 33.1% to 77.1% [14, 23, 24]”.

--Line 332-333, “omitting and introducing a primary outcome were the second and third most common discrepancies” is now corrected as “omitting a primary outcome and reporting the registered secondary outcomes as primary outcomes were the second and third most common discrepancies”.

--Line 358, the sentence is now specified as “as evidenced by our findings revealing that 57.1% of prospectively registered studies with primary outcome discrepancies tended to report statistically significant results”.
